# Dominant-Negative Androgen Receptor Inhibition of Intracrine Androgen-Dependent Growth of Castration-Recurrent Prostate Cancer

**DOI:** 10.1371/journal.pone.0030192

**Published:** 2012-01-17

**Authors:** Mark A. Titus, Brian Zeithaml, Boris Kantor, Xiangping Li, Karin Haack, Dominic T. Moore, Elizabeth M. Wilson, James L. Mohler, Tal Kafri

**Affiliations:** 1 Department of Urology, Roswell Park Cancer Institute, Buffalo, New York, United States of America; 2 Department of Urology, School of Medicine and Biotechnology, University at Buffalo, Buffalo, New York, United States of America; 3 UNC Gene Therapy Center, School of Medicine, University of North Carolina, Chapel Hill, North Carolina, United States of America; 4 Laboratories for Reproductive Biology, School of Medicine, University of North Carolina, Chapel Hill, North Carolina, United States of America; 5 Lineberger Comprehensive Cancer Center, School of Medicine, University of North Carolina, Chapel Hill, North Carolina, United States of America; 6 Department of Pediatrics, School of Medicine, University of North Carolina, Chapel Hill, North Carolina, United States of America; 7 Department of Biochemistry and Biophysics, School of Medicine, University of North Carolina, Chapel Hill, North Carolina, United States of America; 8 Department of Microbiology and Immunology, School of Medicine, University of North Carolina, Chapel Hill, North Carolina, United States of America; National Cancer Institute, United States of America

## Abstract

**Background:**

Prostate cancer (CaP) is the second leading cause of cancer death in American men. Androgen deprivation therapy is initially effective in CaP treatment, but CaP recurs despite castrate levels of circulating androgen. Continued expression of the androgen receptor (AR) and its ligands has been linked to castration-recurrent CaP growth.

**Principal Finding:**

In this report, the ligand-dependent dominant-negative ARΔ142–337 (ARΔTR) was expressed in castration-recurrent CWR-R1 cell and tumor models to elucidate the role of AR signaling. Expression of ARΔTR decreased CWR-R1 tumor growth in the presence and absence of exogenous testosterone (T) and improved survival in the presence of exogenous T. There was evidence for negative selection of ARΔTR transgene in T-treated mice. Mass spectrometry revealed castration-recurrent CaP dihydrotestosterone (DHT) levels sufficient to activate AR and ARΔTR. In the absence of exogenous testosterone, CWR-R1-ARΔTR and control cells exhibited altered androgen profiles that implicated epithelial CaP cells as a source of intratumoral AR ligands.

**Conclusion:**

The study provides *in vivo* evidence that activation of AR signaling by intratumoral AR ligands is required for castration-recurrent CaP growth and that epithelial CaP cells produce sufficient active androgens for CaP recurrence during androgen deprivation therapy. Targeting intracrine T and DHT synthesis should provide a mechanism to inhibit AR and growth of castration-recurrent CaP.

## Introduction

Prostate cancer (CaP) is the most common non-skin cancer diagnosed in American men. Despite earlier detection and improved treatment, more than 33,000 deaths are anticipated in 2011 [1]. For decades, androgen deprivation therapy has been the preferred treatment for locally advanced or metastatic CaP. Androgen deprivation therapy is effective initially but remissions are temporary. CaP that recurs responds poorly to most treatments and almost all men succumb to the disease. A molecular role for the androgen receptor (AR) in the transition to castration-recurrent CaP is supported by the continuous expression of AR [2]–[4] and androgen-regulated genes [5].

Many mechanisms contribute to AR transactivation despite castrate levels of circulating testicular androgens (reviewed by Feldman [6]). However, other studies including our own have demonstrated that castration-recurrent CaP maintains tissue levels of dihydrotestosterone (DHT) sufficient to activate AR [4], [7]–[9]. Persistent tissue testosterone (T) and DHT during androgen deprivation therapy may derive from adrenal androgens, such as dehydroepiandrosterone (DHEA) and androstenedione [7], from androstanediol through the backdoor pathway of DHT synthesis [10], [11] and/or *de novo* production from cholesterol [12]. In castration-recurrent CaP, AR induces the androgen-dependent expression of prostate-specific antigen and the transmembrane protease, serine 2-v-ets erythroblastosis virus E26 oncogene homolog, TMPRSS2∶ERG [13]. A recent report demonstrated that 70% of men with castration-recurrent CaP responded to abiraterone acetate, a cytochrome P450 17α-hydroxylase/lyase (CYP17A1) androgen biosynthesis inhibitor. The antitumor activity of abiraterone acetate in clinical studies suggests that inhibition of cytochrome P450 17α-hydroxylase/lyase (CYP17A1) decreases ligand-activated AR signaling in castration-recurrent CaP [14].

CWR-R1 cells used in the present study derive from the castration-recurrent CWR22 xenograft [15], express the AR-H874Y mutant [16] and proliferate in an androgen-deprived environment. AR is comprised of an NH_2_-terminal transactivation domain, a central DNA binding domain, and a carboxyl-terminal ligand-binding domain (LBD) [17]. AR is full-length in CWR-R1 cells derived from the CWR22 human prostate cancer xenograft, but is susceptible to proteolytic degradation during extraction to a major ∼80 kDa form [18]. AR transcriptional activity is mediated by activation functions in the NH_2_-terminal and LBD. A unique property of AR is that T or DHT induce an NH_2_- and carboxyl-terminal (N/C) interaction [19] mediated by the NH_2_-terminal FXXLF motif [20] that slows ligand dissociation and AR degradation. A role of the NH_2_-terminal domain in gene regulation [21]–[24] has also been reported for non-genomic AR signaling [25].

In the present study, CWR-R1 cells were engineered using lentivirus to overexpress the human AR with a deletion of NH_2_-terminal activation residues 142–337 that results in a transcriptionally inactive dominant negative AR. The human AR deletion mutant ARΔ142–337 (ARΔTR) binds ligand with high affinity but is transcriptionally inactive in the absence or presence of androgen [26]. The mechanism of dominant negative activity is heterodimerization with endogenous AR to prevent transactivation of target genes [27]. The results suggest that intratumoral T and DHT synthesis induces dominant negative ARΔTR inhibition AR dependent CWR-R1 tumor growth. The androgen profile of ΔTR-transduced CWR-R1 tumors in the absence of T support the hypothesis that epithelial CaP cells produce AR ligands in a murine model with low to undetectable adrenal androgen.

## Results

### ARΔTR inhibits AR transactivation and CWR-R1 cell proliferation

**Figure 1:**
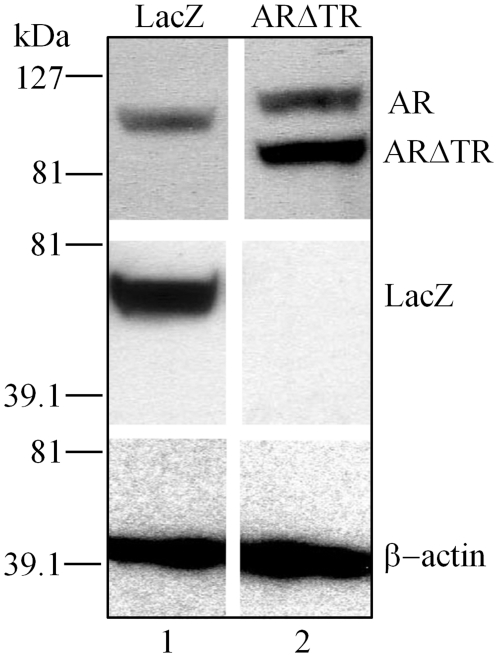
Overexpression of ARΔTR or LacZ transgene in lentiviral vector transduced CWR-R1 cells. Western blot analysis of CWR-R1 protein lysates (20 µg) demonstrated that endogenous AR (M_r_ 110 kDa, lanes 1–2) is detected in both ARΔTR and LacZ transduced CWR-R1 cells using AR goat polyclonal antibody. LacZ (M_r_ 60.5 kDa, lane 1) expression is only observed in LacZ-transduced CWR-R1 cells using LacZ rabbit polyclonal antibody and ARΔTR (M_r_ 84 kDa, lane 2) expression is detected using AR goat polyclonal antibody in ARΔTR-transduced CWR-R1 cells. Endogenous β-actin was detected using β-actin AC-15 mouse monoclonal antibody (M_r_ 43 kDa, lanes 1 and 2) and served as the loading control for both ARΔTR and LacZ-transduced CWR-R1 cells.

**Figure 2:**
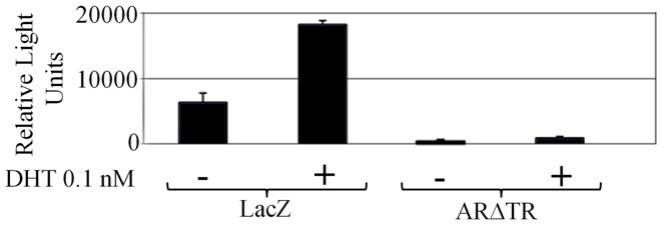
Endogenous AR transcriptional activity in LacZ and ARΔTR lentiviral vector transduced CWR-R1 cells. CWR-R1 cells were transiently transfected with the MMTV-luciferase reporter and assayed for luciferase activity in the presence and absence of DHT. In the presence of 0.1 nM DHT, CWR-R1 cells expressing the LacZ transgene demonstrated an increase in MMTV-luciferase reporter activity compared to control cells without DHT. Expression of ARΔTR reduced MMTV-luciferase reporter activity in the absence or presence of 0.1 nM DHT compared to LacZ-transduced control cells. Columns represent mean luciferase activity in relative light units from 3 independent experiments; bars ± standard deviation.

The effect of intracrine androgen synthesis and ARΔTR inhibition of endogenous AR and castration-recurrent CaP growth was determined in CWR-R1 cells derived from the castration-recurrent CWR22 human CaP xenograft. CWR-R1 cells transduced with lentivirus expressing ARΔTR or LacZ under control of the CMV promoter exhibited high expression (**Fig. 1**). The efficacy of ARΔTR inhibition of AR transcriptional activity was determined using MMTV-Luc transfected into lentivirus-transduced CWR-R1 cells in the absence and presence of 0.1 nM DHT because the prostate-specific antigen-luciferase reporter gene is only weakly activated by endogenous AR [10], [21], [28]. Addition of 0.1 nM DHT LacZ-transduced CWR-R1 cells increased luciferase activity by 3-fold (**Fig. 2**). Under the same conditions, luciferase activity in ARΔTR-transduced CWR-R1 cells was low with or without 0.1 nM DHT, an androgen concentration that maximally stimulates androgen-regulated genes in CWR-R1 cells [29]. The results demonstrate a dominant negative effect of ARΔTR on endogenous AR transactivation of MMTV-luc in the absence and presence of DHT.

**Figure 3:**
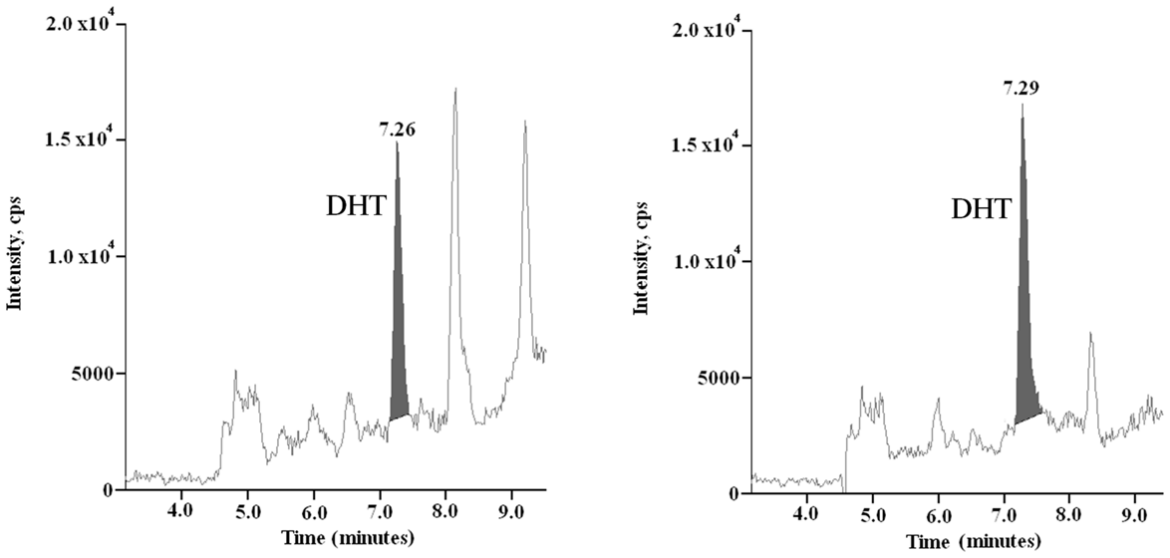
Intracrine levels of DHT in CWR-R1 cells. DHT was measured in CWR-R1 cells grown without media supplements or exogenous T using liquid chromatography tandem mass spectrometry. Representative chromatogram of DHT (left panel, DHT peak at 7.26 min) using 10 million CWR-R1 cells (n = 4). The mean concentration of DHT was 1.84±0.16 fmol/million CWR-R1 cells. Control calibrator chromatogram of DHT standard showing peak at 7.29 min (right panel).

Inhibition of endogenous AR transactivation by ARΔTR in the absence of added DHT raised the possibility that endogenous AR ligands induced ARΔTR dimerization [30] and heterodimerization between ARΔTR and full-length endogenous AR [31]. Liquid chromatography tandem mass spectrometry analysis demonstrated 3.38±0.26 fmol T/million cells (n = 4) and 1.84±0.16 fmol DHT/million cells (n = 4) (**Fig. 3**). Since AR dimerization and DNA binding are androgen-dependent [30], the results suggested that ARΔTR inhibitory activity can serve as a surrogate indicator for intracellular active androgens.

**Figure 4:**
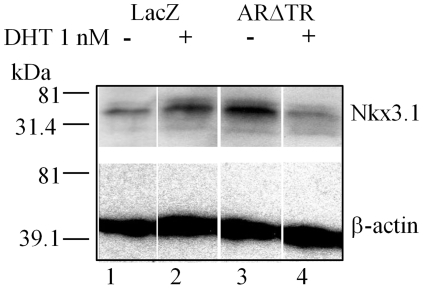
Expression level of androgen-regulated Nkx3.1 protein in CWR-R1 cells expressing LacZ or ARΔTR transgene. Immunoblot analysis of CWR-R1 cell protein lysates (20 µg) determined Nkx3.1 protein expression using goat polyclonal antibody in the presence or absence of 1.0 nM DHT. Nkx3.1 (M_r_ 35.5 kDa) protein was increased in LacZ-transduced CWR-R1 cells with addition of DHT (lanes 1 and 2). ARΔTR-transduced CWR-R1 cells exhibited decreased Nkx3.1 protein expression in the presence of DHT (lanes 3 and 4). Endogenous β-actin (M_r_ 43 kDa) was used as loading control (lanes 1–4).

**Figure 5:**
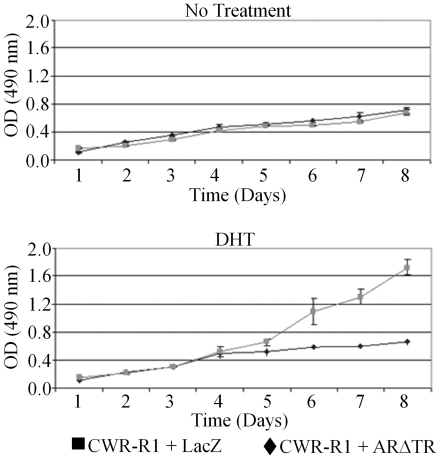
ARΔTR-transduced CWR-R1 cells demonstrate decreased proliferation in the presence of DHT. Cell proliferation was determined using XTT^R^ assay and monitoring absorbance at 490/690 nm in triplicate from day 1 to day 8 (mean ± standard deviation). CWR-R1 cells expressing ARΔTR (solid diamond and black line) or LacZ (solid square and grey line) transgene in the absence of 0.1 nM DHT showed similar growth curves (upper panel). In the presence of 0.1 nM DHT, ARΔTR-transduced CWR-R1 cell growth decreased compared to LacZ-transduced control CWR-R1 cells (lower panel).

The effect of ARΔTR on androgen-dependent Nkx3.1 gene expression [32] was assessed further to characterize the inhibitory activity of ARΔTR on AR signaling. Addition of DHT decreased Nkx3.1 protein levels in ARΔTR-transduced CWR-R1 cells (**Fig. 4**) but increased Nkx3.1 protein in LacZ-transduced CWR-R1 control cells. Addition of DHT increased CWR-R1-LacZ cell proliferation, but there was no increase in ARΔTR-transduced CWR-R1 cell proliferation. In the absence of added DHT, both LacZ- and ARΔTR-transduced CWR-R1 cells exhibited minimal proliferation (**Fig. 5**).

### ARΔTR inhibits CWR-R1 tumor growth

**Figure 6:**
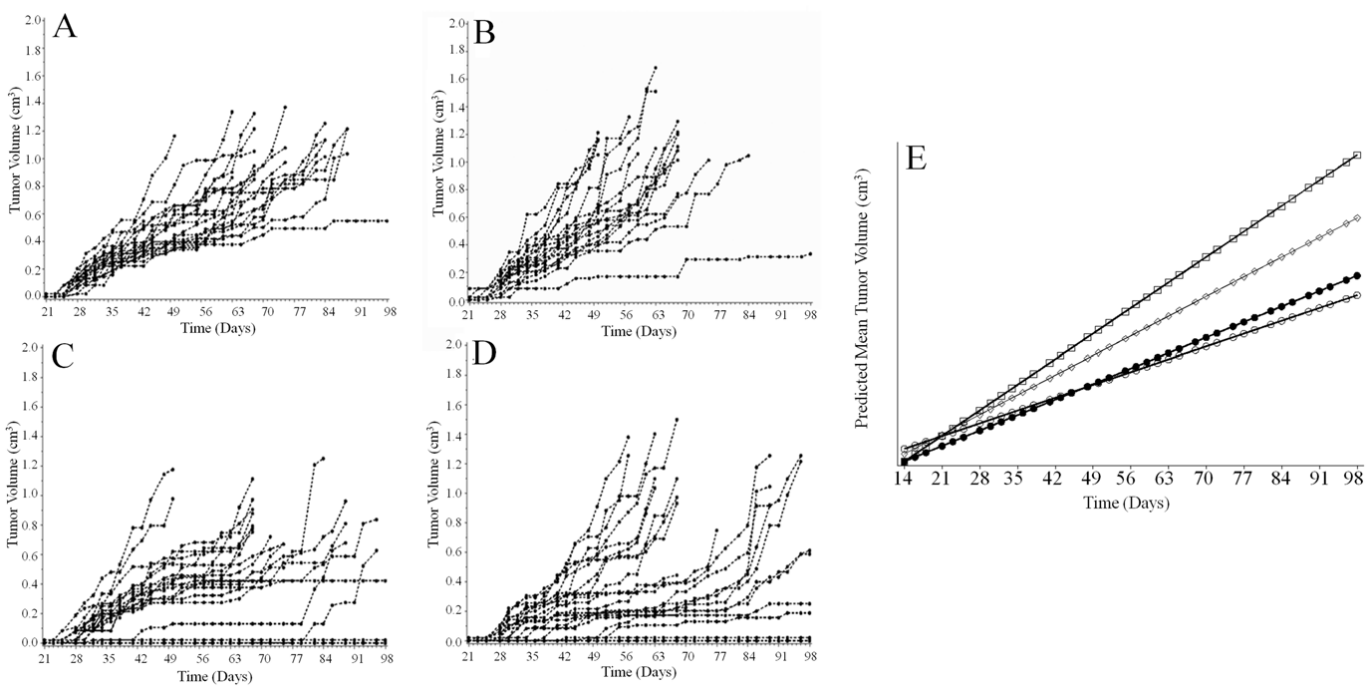
Individual mouse CWR-R1 tumor volume (cm^3^) trajectories and predicted mean tumor volumes from each cohort. Tumors were measured using digital calipers and volumes calculated from day 14 to day 160 after CWR-R1 cell inoculation. Tumor volumes, LacZ (A, n = 22), LacZ+T (B, n = 21), ARΔTR (C, n = 21) or ARΔTR+T (D, n = 21), are shown from day 21 when growth began through day 96 beyond which only 1 mouse in each group remained. Mixed modeling statistical analysis showed a statistically significant difference among the 4 groups. ARΔTR-transduced CWR-R1 tumor growth rates were reduced compared to LacZ controls in the absence (p = 0.01, panel A vs. C) or presence (p = 0.0004, panel C vs. D) of T. (E) Predicted mean CWR-R1 tumor volumes (cm^3^) were plotted against time of first tumor harvest for the LacZ+T (open squares), LacZ (open diamonds), ARΔTR+T (solid circles) and ARΔTR (open circles) groups. ARΔTR-expressing CWR-R1 tumors (circles) exhibit decreased growth rates compared to LacZ-transduced CWR-R1 control cells.

LacZ or ARΔTR-transduced cells were injected into nude mice to generate CWR-R1 tumors to assess the impact of ARΔTR on tumor growth and endogenous AR signaling. Tumor growth rates were altered by ARΔTR expression. A rigorous mixed modeling statistical analysis that considered individual tumor volume trajectories demonstrated significant differences among the 4 groups (**Fig. 6**). The growth rate of LacZ controls differed with exogenous T (p = 0.04) (**Fig. 6A and 6B**), which indicated different controls were required for the two ARΔTR experimental groups. ARΔTR reduced tumor growth rates compared to controls, an effect that was similar without (p = 0.01) or with exogenous T (p = 0.0004) (**Fig. 6C and 6D**). Results were similar when tumor growth was assessed using two additional parameters. LacZ and LacZ+T controls differed by slope (p = 0.01) and doubling time (p = 0.02), which confirmed the need for different controls for each ARΔTR experimental group. T supplements to optimize ARΔTR function decreased tumor growth compared to controls by slope (p = 0.004) and doubling time (p = 0.0007). Without T supplements, tumor growth slope (p = 0.07) and doubling time (p = 0.37) were similar. Inspection of the individual tumor trajectories and actual tumor volumes suggested that the effect of ARΔTR was a combination of slowing the rate of growth and delaying the onset of tumor growth, and that this effect occurred more often when exogenous T is provided to enhance the effect of ARΔTR (**Fig. 6E**).

**Figure 7:**
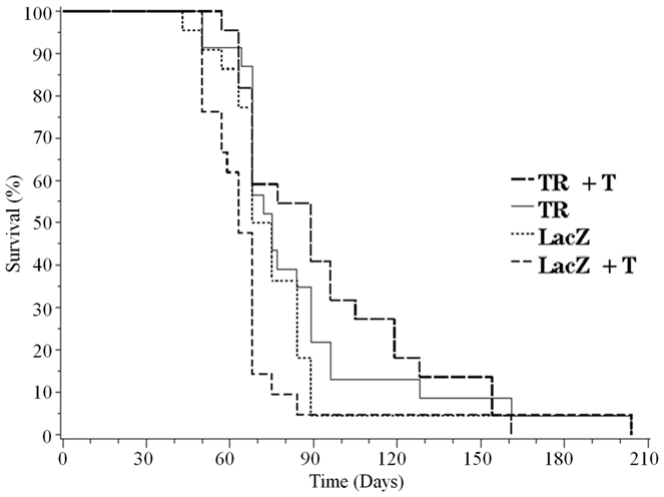
Kaplan-Meier plots from each cohort. Day of euthanasia was based on 1.5 cm^3^ tumor size for mice in the LacZ+T (dashed line, n = 21), LacZ (dotted line, n = 22), ARΔTR (solid line, n = 21) or ARΔTR+T (broken line, n = 21) groups. The median survival time differed in groups that received exogenous T (broken line vs. dashed line, p = 0.008) but not in groups without T (solid line vs. dotted line, p = 0.34).

ARΔTR-induced changes in tumor growth rate also affected the time of euthanasia determined by tumor size exceeding 1.5 cm^3^ (**Fig. 7**, Kaplan-Meier plot; **Table 1**, Log rank analysis, p = 0.009). 95% confidence intervals for the mean time to euthanasia in days and the range for each group were LacZ (71, 68–84 days), LacZ+T (63, 57–68 days), ARΔTR (75, 68–89 days), and ARΔTR+T (89, 68–105 days). ARΔTR increased the time to host median euthanasia by 36% in the presence of exogenous T, but had little effect without exogenous T. Median time to euthanasia increased by 5% in the absence of exogenous T, a difference that was not statistically significant.

**Table 1:** Log-Rank Test p-values in Order of Stepdown Pair-Wise Comparisons of Overall Survival Family-Wise Error Rate (FWR) Controlled p-values.

Comparison		log-rank test p-value
LacZ+T	LacZ	0.03
LacZ+T	ARΔTR+T	0.008
LacZ	ARΔTR	0.34
ARΔTR	ARΔTR+T	0.59
Overall	0.009	

### Negative selection of ARΔTR in CWR-R1 tumors

**Figure 8:**
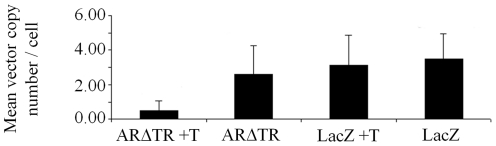
ARΔTR and LacZ mean vector copy number/cell in harvested CWR-R1 tumors with and without T. Vector copy number per cell was determined using quantitative PCR of the Woodchuck hepatitis virus post-transcriptional regulator element. The mean vector copy number per cell is 0.49±0.58 for ARΔTR+T, 2.58±1.68 for ARΔTR, 3.10±1.77 for LacZ+T and 3.48±1.45 for LacZ tumors. A statistically significant decrease in vector copy number per cell was observed for ARΔTR+T vs ARΔTR tumors (p = 0.001) but not LacZ+T vs. LacZ (p = 0.4) tumors.

The similar time to euthanasia based on tumor size between CWR-R1-ARΔTR and LacZ mice in the absence of androgen suggested that ARΔTR cells select against ARΔTR expression. To test this, the ARΔTR and LacZ vector copy number and protein expression were determined in CWR-R1 tumors. ARΔTR or LacZ vector copy numbers and protein expression were determined in transduced CWR-R1 cells prior to inoculation and at the time of tumor harvest. ARΔTR or LacZ vector copy number per cell measured in CWR-R1-transduced cells prior to inoculation based on PCR amplification of the Woodchuck hepatitis virus post-transcriptional regulator element was 5.46 and 4.53, respectively. Mean (± standard deviation) vector copy number per cell for ARΔTR and LacZ-transduced CWR-R1 tumors at the time of harvest was 0.49±0.58 for ARΔTR+T and 2.58±1.68 for ARΔTR (p = 0.001), and 3.10±1.77 for LacZ+T and 3.48±1.45 for LacZ (p = 0.4) (**Fig. 8**).

**Figure 9:**
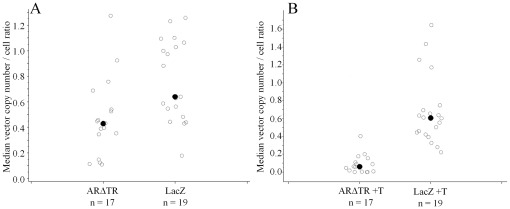
Median vector copy number/cell ratio of harvested CWR-R1 tumor cells versus CWR-R1 cells prior to inoculation. The ratios for (A) ARΔTR and LacZ, and (B) ARΔTR+T and LacZ+T were calculated using the vector copy number measured at time of tumor harvest versus the vector copy number measured prior to inoculation. Individual tumor vector copy number per cell ratio from each cohort is represented using open circles. The median vector copy number ratio (solid circle) was determined in ARΔTR (0.43), LacZ (0.64), ARΔTR+T (0.05), and LacZ+T (0.6) tumors. The ARΔTR transgene was selected against in the absence (p = 0.006) and presence (p<0.0001) of exogenous T compared to LacZ controls.

To characterize further the effect of ARΔTR on vector copy number, ratios of vector copy number per cell in CWR-R1 cells were calculated at the time of tumor harvest and at the time prior to CWR-R1 cell inoculation for ARΔTR and LacZ tumors in the presence and absence of T. Statistical analysis of median vector copy number per tumor cell ratios for ARΔTR and LacZ expressing vectors showed that the ARΔTR expression vector cassette was selected against (p = 0.006, **Fig. 9A**). A similar statistical result was obtained in the median vector copy number per tumor cell ratio for ARΔTR and LacZ expressing vectors in the presence of T (p<0.0001, **Fig. 9B**). Overall, ARΔTR-transduced CWR-R1 cells in the presence or absence of exogenous T selected against the ARΔTR vector compared to LacZ vector controls.

**Figure 10:**
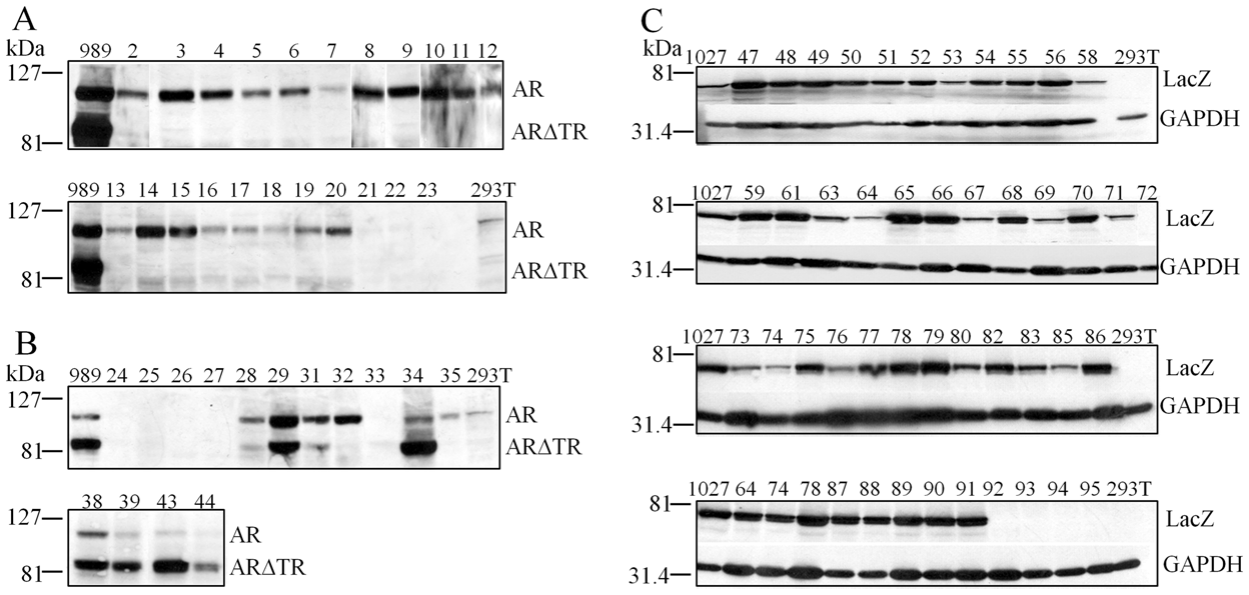
ARΔTR and LacZ protein expression in CWR-R1 tumors with or without exogenous T. Western blot analysis was performed using protein lysates isolated from CWR-R1 tumors collected at the time of tumor harvest. The CWR-R1 tumor expression of endogenous AR (M_r_ 110 kDa), and transduced ARΔTR (ARΔ142–337, M_r_ 84 kDa) and LacZ (M_r_ 60.5 kDa) protein were evaluated. (A) Immunoblots of AR and ARΔTR in the presence of T (tumor numbers 2–23) or (B) absence of T (tumor numbers 24–29, 31–35, 38, 39, 43 and 44). (C) Immunoblots of LacZ (M_r_ 60.5 kDa) protein with T (tumor numbers 47–68) or without T (tumor numbers 69–91) and in empty vector controls (tumor numbers 92–95). LacZ was expressed in all but one of 42 CWR-R1 tumors. LacZ protein expressed in pTK1027 transduced CWR-R1 cells prior to subcutaneous inoculation in mice was used for control. HEK-293T cells were used as negative controls and the loading control for LacZ immunoblots was glucose 6-phosphate dehydrogenase (GADPH, M_r_ 36 kDa).

The altered median ARΔTR and LacZ vector copy number per tumor cell ratios suggested decreased ARΔTR protein expression compared to LacZ controls. Harvested CWR-R1 tumors with sufficient amounts of tissue (79 tumors) for analysis of endogenous AR and expressed ARΔTR or LacZ protein were analyzed on immunoblots. Expression of endogenous AR and ARΔTR protein was demonstrated in pTK989-ARΔTR transduced CWR-R1 cells prior to subcutaneous inoculation. ARΔTR-transduced CWR-R1 tumors in the presence of T (**Fig. 10A**), expressed endogenous AR in all 22 CWR-R1-ARΔTR+T tumors, although AR protein was low to undetectable in 3 samples (samples 21–23). In contrast, ARΔTR protein was negligible or absent in all CWR-R1-ARΔTR tumors in the presence of T supplements.

In the absence of supplemental T, AR protein persisted in 10 of 15 CWR-R1-ARΔTR tumors (tumor numbers 28, 29, 31, 32, 34, 35, 38, 39, 43 and 44), and endogenous AR was not detected in 5 tumors that also did not express ARΔTR (samples 24–27 and 33) (**Fig. 10B**). CWR-R1-ARΔTR tumors 32 and 35 without the T supplement expressed AR but not ARΔTR. ARΔTR was co-expressed in 8 CWR-R1 tumors (tumor numbers 28, 29, 31, 34, 38, 39, 43 and 44).

In contrast, LacZ protein was expressed in all tumors except tumor 72 in the absence and presence of T (**Fig. 10C**). Empty vector control CWR-R1 tumors with T (samples 92 and 93) and without T (samples 94 and 95) and 293T cells did not express LacZ or ARΔTR protein. The consistent expression of LacZ protein corresponded with vector copy number calculated in LacZ-transduced tumors.

The results suggest negative selection of ARΔTR is not limited to CWR-R1-ARΔTR tumors propagated in mice with supplemental T. In ARΔTR-transduced tumors without supplemental T, ARΔTR selection was less possibly due to low serum T and/or altered intracrine T biosynthesis.

### Intracrine synthesis of active androgens in castration-recurrent CaP

The absence of CYP17A1 in mouse adrenals [33] provides a model to test whether castration-recurrent CaP produces intracrine androgens. Generation of the CWR-R1-ARΔTR tumors provided an opportunity to investigate an effect of AR signaling on intracrine active androgen biosynthesis. The similar CWR-R1-ARΔTR tumor growth, tumor doubling times and slopes with and without T, and measurements of DHT in CWR-R1 cells suggested that CWR-R1 tumors produced active androgens in the absence of circulating T.

To identify possible differences in androgen profiles between CWR-R1-ARΔTR and LacZ tumors, T, DHT, androstenedione and androsterone were measured using liquid chromatography tandem mass spectrometry. Tissue levels of 0.32 nM DHT (p = 0.59) and 0.05 nM androsterone (p = 0.23) measured at the time of tumor harvest were similar in ARΔTR and LacZ-transduced tumors (**Table 2**) and sufficient to activate endogenous AR and ARΔTR (see *in vitro* results; **Fig. 2**). Furthermore, 3.25 nM T in LacZ tumors was similar to T levels in castration-recurrent CaP [4], [9] that was sufficient to activate endogenous AR-H874Y [29]. However, in CWR-R1-ARΔTR tumors, T levels were ∼4-fold less than LacZ tumors, which suggested alteration in T biosynthesis by the dominant negative AR. Decreased T biosynthesis in ARΔTR-transduced CWR-R1 tumors correlated with the accumulation of ∼1 nM androstenedione, an androgen precursor to T. In contrast, CWR-R1-LacZ tumors contained 1.05 nM androstenedione. Quantitation of 5α-reduced and unsaturated androgen precursors in ARΔTR and LacZ-transduced CWR-R1 tumors suggested intracrine androgen biosynthesis contributed to AR-dependent castration-recurrent CaP growth.

**Table 2:** Mean androgen levels (nM, ± SEM) measured in LacZ (n = 8) and dominant-negative ARΔTR (n = 11) CWR-R1 androgen-independent tumors without exogenous T pellets.

	Androstenedione	Testosterone	Dihydrotestosterone	Androsterone
LacZ	1.05±0.18	3.25±0.37	0.28±0.17	0.07±0.02
ARΔTR	13.6±4.9	0.79±0.33	0.32±0.11	0.05±0.02

## Discussion

Studies in this report were based on the premise that a dominant negative form of AR with a deletion of the NH_2_-terminal transactivation domain requires androgen for dimerization [30] and inhibition of transcriptional activity of full-length AR [27]. We have shown that stable expression of dominant negative ARΔTR inhibits endogenous AR-H874Y transcriptional activity and slows or delays CWR-R1 tumor growth in the absence and presence of supplemental T. Dominant negative ARΔTR activity was also indicated by the decrease in Nkx3.1 protein and luciferase reporter activity in the presence of DHT.

Results from the study have revealed two additional important findings. First, there was essentially 100% negative selection against the dominant negative form of AR during castration-recurrent CWR-R1 tumor growth in the presence of supplemental T. This contrasted nearly 100% retention of the LacZ control gene. Both dominant negative ARΔTR and LacZ were integrated into the genome using lentivirus expression and cell selection prior to cell inoculation and tumor growth. Second, approximately 50% exclusion of dominant negative AR occurred in CWR-R1-ARΔTR tumors propagated in the absence of supplemental T. These results, together with mass spectrometry measurements of T and DHT in cultured CWR-R1 cells, provide evidence for intracrine synthesis of active androgens needed for AR signaling in CaP. Selective loss of a dominant negative AR demonstrates the genetic versatility of castration-recurrent CaP cells to maximize AR signaling.

### Intracrine synthesis of androgen in castration-recurrent CaP

Inhibition of luciferase activity in CWR-R1 cells by dominant negative ARΔTR in the absence of exogenous T suggested intracellular synthesis of T. This was supported by mass spectrometry measurements of T and DHT in CWR-R1 cells. Our findings are in agreement with previous evidence that androgen-starved LNCaP cells synthesize androgens from ^14^C-acetate labeled cholesterol [34]. CaP cells alter cholesterol metabolism and processing to support androgen biosynthesis [35]. Intracrine biosynthesis of androstenedione, DHEA and T was also demonstrated in CWR-R1 and PC-3 cells, and implicated CYP17A1 activity in AR positive and negative CaP cells [36]. In clinical specimens, cholesterol and androgen biosynthetic enzymes were up-regulated in castration-recurrent CaP [12], [37], [38].

In the CWR-R1-LacZ tumors, intratumoral T and DHT levels were sufficient to activate AR-H874Y and promote tumor growth. Intratumoral DHT levels in ARΔTR and LacZ-transduced tumors were similar. However, in ARΔTR-transduced CWR-R1 xenograft tumors, T levels were lower and androstenedione levels were greater than CWR-R1-LacZ tumors. Lower T levels in the CWR-R1-ARΔTR tumor suggested that dominant negative ARΔTR selected against cells expressing aldo-keto reductase 1C3, an enzyme that reduces androstenedione to T in castration-recurrent CaP, or increased the oxidation of T to androstenedione by NAD^+^ dependent 17β-hydroxysteroid dehydrogenase-10 activity [39]. Decreased conversion of androstenedione to T would shift the intracellular ligand profile toward activation of mutant AR-H874Y from wild-type LBD AR in ARΔTR. Intracrine metabolism of cholesterol to active androgens could explain the initial sensitivity of castration-recurrent CaP to treatment with abiraterone acetate [14].

AR-H874Y endogenous to CWR-R1 cells retains high affinity T and DHT binding similar to wild-type AR. However, the H874Y mutation stabilizes the LBD core structure so that T is as effective as DHT in promoting transcriptional activity [29]. The structure stabilizing effects of H874Y are sufficient to overcome the detrimental effects of loss of function mutations that cause androgen insensitivity [40]. The equivalent potency of T and DHT with AR-H874Y suggests that intracrine synthesis of T would promote LacZ-transduced CWR-R1 tumor growth. Intracellular conversion of androstenedione to T could have a substantial transcriptional effect on AR-H874Y relative to wild-type AR [29]. Increased expression of p160 steroid receptor coactivators in castration-recurrent CaP [41], [42] contributed to the hypersensitization.

T∶DHT ratios in LacZ- and ARΔTR-transduced CWR-R1 tumors were similar to those in castration-recurrent CaP [4], [9], [43]. Previous studies using clinical specimens demonstrated decreased 5α-reductase-2 isozyme mRNA [44] and protein [45] expression in castration-recurrent CaP that was partially due to loss of secreted stromal cell factor signaling [44], [46]. CWR-R1 tumors also may have decreased 5α-reductase-2 activity. The similar DHT levels in LacZ and ARΔTR tumors indicate that DHT biosynthesis depends on androstenedione rather than T, since androstenedione is a better substrate for 5α-reductase-1 than T [47]. The shift toward conversion of androstenedione to androstanedione [45] by 5α-reductase-1 and further metabolism to DHT by membrane bound NADPH dependent 17β-hydroxysteroid dehydrogenase-15 [48] may explain why men with castration-recurrent CaP and high baseline androstenedione levels survived longer when treated with ketoconazole [49]. DHT levels remain similar even though the T∶androstenedione ratios in LacZ and ARΔTR tumors were inversed. The low and similar DHT levels (∼10^−11^) measured in LacZ and ARΔTR-transduced CWR-R1 tumors may be the optimum intratumoral concentration for CWR-R1 cell proliferation [42] and AR-H874Y coordinated DNA replication licensing [50].

### Negative selection to enhance AR activity

The growth inhibitory effects of ARΔTR were complicated by the fact that the ARΔTR transgene was negatively selected against during CWR-R1 tumor growth most efficiently in the presence of supplemental T. Expression of the ARΔTR transgene was lost in all of the 22 ARΔTR+T tumors by the time of harvest, while the ARΔTR transgene was retained in ∼50% of tumors in the absence of supplemental T. The mean tumor volume was larger than ARΔTR+T tumors, which may be associated with a partial switch from T to androstenedione biosynthesis by these tumors. Although the timing of negative selection against the ARΔTR transgene was not rigorously tested, preliminary studies suggested loss of the transgene occurred approximately 10 days after tumor cell inoculation on day 23.

Negative selection of the ARΔTR transgene was supported by the decreased genome vector copy number in both ARΔTR and LacZ-transduced CWR-R1 tumors in the absence or presence of exogenous T. Loss of transgene expression has been shown to occur 10 to 15 days after lentiviral transduced embryonic carcinoma cells [51]. The delay in ARΔTR+T tumor growth supported selection against the transgene after 10 days in the CWR-R1 model. It could be argued that loss of the transgene resulted from vector escape from variegation and/or extinction events. On the other hand, random gene or chromosomal deletion and transgene silencing [51] were not supported, as there was nearly complete retention of the control LacZ transgene in the CWR-R1 tumors.

Recent studies have demonstrated that CWR-R1 cells, as well as the parental CWR22 CaP xenografts, contain replication competent retroviruses identical to the xenotropic murine leukemia related virus (XMRV) found in human CaP cells [52], [53]. XMRV evolved by recombination between two endogenous retroviruses in nude mice carrying the CWR22 xenografts [52], [53]. XMRV proviruses were not detected in samples obtained from early CWR22 tumor passages, which is consistent with newer reports that XMRV is not involved in the development of CaP in humans. The data presented herein does not exclude the unlikely possibility that XMRV infection contributes to castration-independence of CWR-R1 cells (derived from CWR22) [53]; Paprotka et al. found this scenario most unlikely. The experiments were well controlled and XMRV infection was present in experimental and control animals. Overall, the results presented here demonstrate inhibition of the CWR-R1 tumor growth by dominant negative inhibition of AR signaling, and indicate the central role of tumor-derived androgens in the development of castration-resistant CaP. The decrease and delay in castration-recurrent tumor growth, coupled with a 36% extension of survival in the presence of T, supported the importance of inhibition of AR expression/activity as a clinical target [54], [55].

## Materials and Methods

### Generation of ARΔTR and LacZ transduced CWR-R1 cells

**Figure 11:**
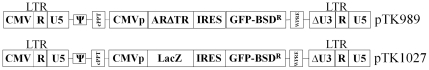
Dominant-negative androgen receptor (ARΔTR, pTK989) and β-galactosidase (LacZ, pTK1027) lentiviral expression vector diagrams. The residual HIV-1 content of both vectors includes the long terminal repeat (LTR) regions, packaging signal of HIV-1 (ψ) and the polypurine tract (cPPT). The 5′ CMV promoter and polyadenylation signal in the 5′ LTR direct high level expression of transfer vector genome. The internal expression cassette consists of CMV promoter, respective transgene coding sequences of the dominant-negative human androgen receptor (ΔTR) or β-galactosidase (LacZ), the internal ribosome entry site (IRES) to express green fluorescence protein fused to blasticidin resistance gene (GFP-BSD^R^) and the Woodchuck hepatitis virus post-transcriptional regulatory element. The 3′ LTR contains U3 deleted self-inactivating sequence (ΔU3).

The coding region of pCMV-hARΔ142–337 with wild-type LBD and FXXLF motif binding motif [20] was subcloned into the BamHI site of SK+ plasmid (Stratagene, La Jolla, CA). The plasmid was digested with XhoI/SpeI and the fragment was subcloned into the XhoI/XbaI site of HIV-1 based vector pTK642 upstream of the IRES-GFP-BSD cassette. The resulting vector pTK989 expressed ARΔTR under control of the human cytomegalovirus promoter (**Fig. 11**). LacZ expression vector pTK1027 was generated by ligation of the SmaI/XhoI fragment that contains LacZ cDNA pTK1022 into pTK642 digested with HpaI/XhoI. All plasmid sequences were confirmed using direct sequencing.

HIV-1 based vectors were generated using a transient 3-plasmid transfection method. The standard calcium phosphate protocol was used as described [56]. Briefly, vector constructs (15 µg), vesicular stomatitis virus glycoprotein envelope expression cassette (5 µg) and ΔNRF packaging cassettes (10 µg) were transfected into 293T cells (ATCC #11268). Lentiviral particles were collected 60 h after transfection from filtered conditioned medium. Lentivirus stocks were aliquoted and stored at −80°C. Virus titers were determined by scoring green fluorescent protein expression following serial dilutions on 293T cells and/or by using the p24 ELISA assay (PerkinElmer Life Sciences, Inc., Waltham, MA, catalog #NEK050). CWR-R1 cells (passage 21) propagated as described [15] were tranduced with lentivirus-ARΔTR or lentivirus-LacZ at multiplicity of infection 5 and selected using blasticidin (Invitrogen, Carlsbad CA, 20 µg/mL).

### Lentivirus transduced CWR-R1 endogenous AR transactivation and cell proliferation

Blasticidin-selected ARΔTR or LacZ CWR-R1 cells (10^6^ cells/6 cm dish) were transfected with 0.5 µg mouse mammary tumor virus luciferase (MMTV-Luc) reporter vector using Effectene reagent (Qiagen, Valencia, CA). After transfection, cells were placed in serum-free, phenol red-free medium in the absence or presence of 0.1 nM DHT. Twenty-four h later, medium with or without DHT was exchanged. Luciferase assays were performed the next day using the Luciferase Assay System (Promega, Madison, WI). ARΔTR or LacZ-transduced CWR-R1 cell proliferation assays were performed using the XTT^R^ assay kit (Roche, Indianapolis, IN, catalog #11465015) in the presence and absence of 0.1 nM DHT.

### CWR-R1 cell and tumor immunoblot analysis

AR, ARΔTR, LacZ and Nkx3.1 homeobox protein immunoblot analyses were performed as described [15] using CWR-R1 cells treated with 1 nM DHT or CWR-R1 tumor specimens stored at −80°C. Protein lysates (20 µg) prepared from CWR-R1 cells or pulverized CWR-R1 tumor specimens were separated using 4–8% acrylamide gradient gels containing SDS and electroblotted to nitrocellulose membranes (Nitrobind®, Osmonics, Inc., Minnentonka, MN). Antihuman AR goat polyclonal antibody (Abcam, Cambridge, MA, catalog #ab19066, 1∶1000) targeted AR N-terminal amino acids 2–16. Rabbit polyclonal LacZ antibody (Millipore, Billerica, MA, catalog #AB1211) and rabbit polyclonal GADPH (Santa Cruz Biotechnology, Santa Cruz, CA, catalogue # sc-25778) were used at 1∶1000 dilution. Nkx3.1 goat polyclonal antibody (Santa Cruz Biotechnology, Inc, Santa Cruz, CA, catalog #sc-15021) and β-actin AC-15 mouse monoclonal antibody (Abcam, Inc., catalog #ab6276) were used at 1∶1000 and 1∶5000 dilutions, respectively. Secondary rabbit-antigoat, goat-antirabbit or anti-mouse IgG antibodies conjugated to horseradish peroxidase were used at 1∶10,000 dilution (Pierce, Rockford, IL). Specific signals were detected using enhanced chemiluminescence (Amersham Pharmacia Biotech, Piscataway, NJ).

### CWR-R1 tumor studies

Mice were maintained and experimental procedures performed in accordance with the National Institute of Health, the University of North Carolina School of Medicine Institutional Animal Care and Use Committee and the Institutional Biosafety Committee. Male athymic nude mice 4 to 5 weeks old were purchased from Harlan Sprague-Dawley, Inc. (Indianapolis, IN) and housed individually in the Division of Laboratory Medicine facility at the University of North Carolina School of Medicine. Each mouse was identified using a numbered ear tag. Two days after castration, mice were divided into two groups of 45 mice each. One T pellet (12.5 mg, 90-day release) was implanted subcutaneously in each of 22 mice from each group to normalize circulating levels to ∼4 ng/mL T (Innovative Research, Sarasota, FL). Mice were inoculated subcutaneously on one flank with 1.25×10^6^ CWR-R1 blasticidin-selected cells suspended in Matrigel (1∶1 mixture, BD Biosciences, Bedford, MA). Matrigel was used to promote xenograft formation in the castrate microenvironment of the inoculation site [57]. Mice received cells transduced with lentivirus-ARΔTR or lentivirus-LacZ. The tumor growth suppressing or promoting activity of host innate immune system was accounted for by comparing ARΔTR to control lentivirus-LacZ groups. Tumor volume was measured at least 3 times per week using digital calipers and volume calculated using the equation L_1_×(L_2_)^2^×0.523. Mice were euthanized and tumors excised when tumor volume reached 1.5 cm^3^. Tumors were cut into ∼0.1 cm^3^ pieces with 1/3 frozen in liquid nitrogen and stored at −80°C, 1/3 immersed in RNAlater (Ambion, Austin, TX) for 24 h and stored at −80°C, and 1/3 fixed in 10% formalin-buffered saline for 24 h, washed in phosphate buffered saline and embedded in paraffin.

### Quantitative PCR

Quantitative PCR of the Woodchuck hepatitis virus post-transcriptional regulatory element was performed to assess ARΔTR and LacZ transgene integration and retention using the 7500 Real Time PCR System and SDSv1.x Software (Applied Biosystems, Foster City, CA). DNA was isolated from RNAlater treated frozen tumor samples and prepared for real-time PCR as described [58]. In brief, tumor DNA were digested with restriction enzyme DpnI to degrade plasmid/vector DNA that remained after transfection. Genomic DNA (10 ng) was normalized using human β-globin gene (2 copies/diploid cell). DNA (1 ng) was calculated equal to 151.5 copies for 1 copy/diploid cell, or 303 copies for 2 copies/diploid cell. An equal β-globin equivalent was used to amplify viral DNA. DNA isolated from FLP9 cells that contained a single copy of provirus per diploid cell was used as a standard [59]. The FLP9 cell line was generated by the Flip-In System (Invitrogen) employing Flip-mediated recombination to introduce a single lentiviral vector genome into HEK293 genome containing a single FRT site. Primers for β-globin gene were forward primer 5′-CAGAGCCATCTATTGCTTAC -3′ and reverse primer 5′-GCCTCACCACCAACTTCATC-3′. Lentivirus specific primers to amplify the WRPE sequence were forward primer 5′-ACGTCCTTCTGCTACGTCC-3′ and reverse primer 5′-AAAGGGAGATCCGACCGACTCGTC-3′. The quantitative PCR reaction contained 1× Master mix (Promega, Madison, WI), 0.08× SYBR® Green I (Cambrex Bio Science, Rockland, ME), 300 nM forward primer, 300 nM reverse primer and template in a total volume of 15 µL. Quantitative-PCR conditions were 95°C for 5 min; 40 cycles at 95°C for 30 sec; annealing at 55°C for 30 sec; and extension at 72°C for 30 sec. A minus template incubation was used as control. An aliquot (10 µL) of each PCR reaction was subjected to gel electrophoresis. PCR products were visualized using ethidium bromide staining. Each experiment was performed 3 times.

### Androgen measurements in CWR-R1 tumors

Tumor samples stored at −80°C were pulverized using liquid nitrogen. Tissue was transferred into high pressure liquid chromatography grade water (50 mg/mL) containing deuterated internal standard, 5α-androstan-17β-ol-3-one-16,16,17-d_3_ (1.0 ng, CDN isotopes, Pointe-Claire, Quebec, Canada). Samples were homogenized and extracted 3 times with 1 mL chloroform∶acetone (9∶1). Organic extracts were combined and dried. Androgens were purified and concentrated using solid phase C18 extraction cartridges (Varian, Palo Alto, CA). Liquid chromatography tandem mass spectrometry was performed as described [9] using the AB SCIEX Triple Quad™ 3000 system (Applied Biosystems, Foster City, CA) with modification. Steroids were eluted using a linear gradient of 65% to 80% mobile phase A (0.4 mM ammonium formate) for 2.25 min and mobile phase B (0.4 mM ammonium formate in methanol) followed by isocratic elution at 95% mobile phase A for 13 min at flow rate 175 µL/min at 60°C. A Phenomenex Luna C18 column (3.0 µm, 150×2 mm) was used to separate T, DHT, androstenedione and androsterone. The parent–product positive ion pairs monitored (mass to charge ratio) were 289.2 to 97.0 for T, 291.2 to 255.2 for DHT, 294.2 to 258.2 for 5α-androstan-17β-ol-3-one-16,16,17-d_3_ internal standard, 287.2 to 97 for androstenedione and 291.2 to 255.2 for androsterone.

### Statistical Methods

Tumor volumes were measured serially and analyzed using random coefficient modeling beginning on day 14 after cell inoculation until the tumor volume exceeded 1.5 cm^3^ when the mice were euthanized (the longest was day 204). The primary interest of this analysis was to investigate the pair-wise difference in growth rates of CWR-R1-ARΔTR, ARΔTR+T, LacZ and LacZ+T tumors. This approach was chosen *a priori* that used a step-down approach to control the family-wise error rate for statistical comparisons of interest. The nonparametric Wilcoxon rank-sum test (with Van der Waerden normal scores) was used for each of the pair-wise 2-group comparisons, when summary measures of slopes and doubling times were compared. The Kruskal-Wallis test (with Van der Waerden normal scores) was used when more than 2 groups were compared. These comparisons yielded essentially equivalent results to parametric random coefficient modeling. The Kaplan-Meier method was used to estimate the time to euthanasia function for each of the 4 groups. The log-rank test was used to test for differences among survival curves. Statistical analyzes were performed using SAS statistical software, version 9.2 from the SAS Institute, Inc., Cary, NC.
